# Assessing musculoskeletal examination skills and diagnostic reasoning of 4th year medical students using a novel objective structured clinical exam

**DOI:** 10.1186/s12909-016-0780-4

**Published:** 2016-10-14

**Authors:** R. Brent Stansfield, Lisa Diponio, Cliff Craig, John Zeller, Edmund Chadd, Joshua Miller, Seetha Monrad

**Affiliations:** 1 University of Michigan Medical School, 1500 E Medical Center Dr., Ann Arbor, MI 48109 USA; 2 1560 E. Maple Rd, Troy, MI 48083 USA; 3 Wayne State School of Medicine, Detroit, MI USA

**Keywords:** Physical examination, Diagnostic reasoning, Skills assessment, Undergraduate medical students, Musculoskeletal

## Abstract

**Background:**

Medical students have difficulty performing and interpreting musculoskeletal physical examinations and interpreting the findings. Research has focused on students' knowledge deficits, but there are few direct assessments of students' ability to perform a hypothesis-driven physical examination (HDPE). We developed a novel musculoskeletal Objective Structured Clinical Exam (OSCE) focusing on HDPE skills for disorders of the shoulder, back and knee, and used it to explore medical student diagnostic reasoning.

**Methods:**

A multidisciplinary group of musculoskeletal specialists developed and gathered validity evidence for a three station OSCE focusing on the HDPE of the shoulder, back and knee, emphasizing the ability to anticipate (identify pre-encounter) expected physical exam findings, and subsequently perform discriminatory physical examination maneuvers. The OSCE was administered to 45 final year medical students. Trained faculty observed and scored students’ ability to anticipate exam findings and perform diagnostic examination maneuvers on simulated patients. Encounters were digitally recorded and scored again by another trained faculty member. Inter-rater reliability for each maneuver was estimated using type-2 intra-class correlations (ICC). Percentages of perfect scores for anticipation and performance were calculated. Pearson’s correlation between anticipation and performance scores was computed for each maneuver and their relationship to diagnostic accuracy was tested with logistic regression.

**Results:**

Inter-rater reliability was good (ICC between .69 and .87) for six exam maneuvers. Maneuver performance was overall poor, with no discriminatory maneuver performed correctly by more than two thirds of students, and one maneuver only performed correctly by 4 % of students. For the shoulder and knee stations, students were able to anticipate necessary discriminatory exam findings better than they could actually perform relevant exam maneuvers. The ability to anticipate a discriminatory finding correlated with the ability to perform the associated maneuver correctly, with the exception of the ability to perform maneuvers needed to diagnose a torn anterior cruciate ligament of the knee. Neither the ability to anticipate or perform was predictive of identifying correct diagnoses for the different cases.

**Conclusions:**

A novel musculoskeletal OSCE, based on principles of the hypothesis-driven physical examination, was able to identify significant deficiencies in examination skills needed to diagnose common disorders of the shoulder, back and knee amongst graduating medical students. In addition, the OSCE demonstrated that accurate anticipation of discriminatory examination findings correlates with ability to perform the associated maneuver; however, the ability to anticipate exceeds the ability to perform. Students do not appear to be using the physical exam to inform their diagnostic reasoning. The findings of this study have implications for both assessment and teaching of the musculoskeletal exam.

**Electronic supplementary material:**

The online version of this article (doi:10.1186/s12909-016-0780-4) contains supplementary material, which is available to authorized users.

## Background

The burden of musculoskeletal disease in the United States is enormous [1] and the physician workforce must receive adequate and appropriate training to meet this growing need. While recent trends in medical school musculoskeletal education have been encouraging [2, 3] there remains evidence for significant deficits. Many studies at different institutions have demonstrated that students have low confidence in their ability to perform a musculoskeletal physical exam [4, 5]. Importantly, national performance on the USMLE Step 2 Clinical Skills Examination shows that musculoskeletal physical examination performance is significantly poorer than physical examination performance in most other domains [6]. Clearly, undergraduate medical education must continue improving the quality of musculoskeletal physical examination teaching. However, a better understanding of why students struggle with this examination is needed in order to address the problem.

There is ample evidence for deficits in students’ knowledge base. The most frequently utilized tool in recent years to assess students’ cognitive knowledge of musculoskeletal medicine is the Freedman and Bernstein examination [7]. This 25 question short-answer test has demonstrated lack of basic musculoskeletal knowledge at many institutions and with many levels of learners [4, 5, 8–12]. However, it is unclear whether poor performance on a knowledge test is predictive of poor clinical performance.

Many schools use objective structured clinical examinations (OSCEs) in order to assess clinical skills, and many include musculoskeletal stations. In addition, musculoskeletal-focused OSCEs have been developed for learners of differing background and experience (for example: [13–15]). Standard OSCE procedures ask a student to perform a diagnostic maneuver, or give students a clinical question and require them to perform a diagnostic maneuver to answer the question. However, few of the published OSCEs explicitly describe an educational conceptual framework or a multidisciplinary approach guiding OSCE development. Importantly, it is unclear whether successful performance on an OSCE indicates successful clinical reasoning [16]; to do so requires addition of a specifically designed reasoning task.

The Hypothesis Driven Physical Exam (HDPE) [17] is a learning and assessment procedure developed in response to the decontextualization of early clinical skills learning (ie, head to toe approaches to clinical exam, rather than examinations focused on clinical presentation) and was designed to facilitate assessment of diagnostic reasoning. The HDPE is grounded in research demonstrating that clinical signs are better identified if the correct diagnoses are in mind; that categories of disorders are better learned when initial exposure is limited to a few prototypical members; and that a limited number of key findings can help discriminate between diagnoses [18–20].

In order to perform a diagnostic musculoskeletal physical examination, a student must a) know what to do, and how/when/why to do it; b) be able to do it; and c) interpret the findings. The purpose of our study was to determine whether explicit prompting for a hypothesis (anticipation) regarding a particular clinical problem either improved performance of the relevant diagnostic maneuvers, or improved identification of the correct diagnosis. To that end, we developed a novel OSCE assessing hypothesis-driven diagnostic skills for disorders of the shoulder, back and knee, and administered it to a sample of graduating medical students. The novel elements of this OSCE included: 1) a multidisciplinary approach to OSCE development that incorporated input from three different musculoskeletal specialties, in order to improve validity of the assessment; 2) grounding in a conceptual framework; and 3) a deliberate incorporation of a pre-encounter anticipation step prior to performance of the examination, in order to identify deficits in the diagnostic reasoning process.

## Methods

### OSCE development and implementation

Through an iterative consensus process, a group of musculoskeletal medical educators representing orthopaedic surgery (CC and JZ), physical medicine and rehabilitation (LD) and rheumatology (SM) developed a three station OSCE focusing on common disorders of the shoulder, back and knee (see Table 2). We chose these regions as common sites of musculoskeletal dysfunction that all graduating medical students should be able to evaluate, based on literature review and consensus discussion. OSCE development included selection of potential diagnoses and discriminatory maneuvers; creation of case vignettes; development of checklists and scoring rubrics; and development of simulated patient (SP) performance requirements and training instructions (Additional file 1). Messick’s framework was used to determine evidence for validity [21].

The OSCE was administered as follows. For each region, students were provided with a brief written clinical vignette and a choice of three possible diagnoses to consider. They were prompted to write down anticipated discriminatory physical exam findings and maneuvers for each potential diagnosis, self-assess their ability to perform the maneuvers, and estimate their future need for these exam skills. They then performed a targeted physical exam, without additional history taking, of an SP mimicking clinical features of one of the diagnoses per region [for back, L5 radiculopathy (Radic); for knee, ACL tear (ACL); for shoulder, rotator cuff impingement (Impingement)]. Subsequently, students documented their preferred diagnosis, along with their reason for choosing it. Student performance of discriminatory exam maneuvers was directly observed and scored by trained faculty using a checklist. All encounters were digitally recorded for review and independent scoring by a second trained faculty, who also scored the anticipation.

Anticipation and performance of relevant maneuvers were scored on a 0-2 scale, with zero being completely wrong, one partially correct, and two completely correct. In cases where multiple exam maneuvers were acceptable (e.g. shoulder impingement), performance of only one was required to receive credit. Detailed written descriptions of what constituted correct anticipation and performance of each maneuver were created by the core faculty team and reviewed with all faculty raters, along with rater training on checklist use. To get a perfect anticipation score, a student needed to name or describe each maneuver correctly. To get a perfect performance score, a student needed to visibly perform all elements of each maneuver correctly. Student diagnoses for each region were scored as correct or incorrect. SPs received a one hour training session, and in most cases SPs participated in the same regional stations across all OSCE sessions to maximize reliability of simulation.

### Study participants

IRB exemption was obtained from the institutional review board (study number HUM00054327). Medical students in the final months of their final year of medical school were recruited via email solicitations to the entire class. Incentive in the form of immediate feedback on their clinical skills and a $50 gift card was offered for participation. All student data were anonymized prior to analysis.

### Analysis

Single faculty ratings of student anticipation (A) were used as measures of student hypothesis-driven reasoning. Two faculty ratings of student maneuver performance (P) were used as measures of student physical exam performance skill. Three maneuvers were not scored: there was no diagnostic maneuver for lumbar stenosis, adhesive capsulitis showed too much inter-rater disagreement, and patello-femoral exam performance was not visible on the video and so could not be rated by a second rater. For the remaining six maneuvers, inter-rater reliabilities of faculty ratings of P were estimated using intraclass correlations (ICC), and for ratings with acceptably high ICCs, the mean scores across raters for P were computed for each maneuver. Percentages of students with perfect scores for A and P were also calculated. The descriptive statistics of these measures were examined to measure student performance of A and P and the relative difficulty of the maneuvers.

To examine the relationship of hypothesis-driven reasoning and clinical performance, Pearson’s correlation between A and P mean scores was computed for each maneuver. High correlations indicated that students who correctly anticipate findings were more likely to perform associated maneuvers well. We also compared absolute A and P scores using student’s t-tests; this assessed whether raters felt that students were better at anticipating findings or performing physical exam maneuvers.

Finally, we tested whether students with good anticipation and/or examination skills were more likely to diagnose the cases correctly. For each region, diagnostic accuracy was modeled using logistic regression with student A and P scores for the two appropriate maneuvers as predictors.

### Source of funding

This research was supported by a grant from the Gilbert Whittaker Fund for the Improvement of Teaching, provided by the institution’s Center for Research on Learning and Teaching. Additionally, the American College of Rheumatology - Rheumatology Research Foundation provided salary support for one investigator (SM).

## Results

Forty-five fourth-year students participated in the study just prior to graduating from medical school. Student demographics are shown in Table 1. 43 % of students had some prior elective musculoskeletal experience, whether clinical, research or both. Only 6.6 % of students were planning to specialize in a musculoskeletal field.

**Table 1:** Demographics of the participating student sample

Demographics	
No. students (% of graduating class of 2012)	45 (30.4 %)
Age (years: avg + SD)	26 + 1.4
Women (%)	23 (51 %)
Future specialty	
- Musculoskeletal (Orthopaedics/PM&R)	3 (6.6 %)
- Internal/family/pediatric/emergency medicine	17 (37.7 %)
- Other	25 (55.6 %)
Previous elective musculoskeletal experience	
- None	26 (57.7 %)
- Research only	1 (2.2 %)
- Clinical only	13 (28 %)
- Research and clinical	5 (11.1 %)

Inter-rater reliability of performance scores was acceptably high for the scoring of the six exam maneuvers (see Table 2); thus mean ratings for performance scores across raters were computed for each maneuver. When examining student performance ratings, we found that each region had one maneuver where students performed very poorly and one where they performed less poorly. Descriptive statistics of performance ratings are found in Table 2.

**Table 2:** Descriptive statistics of performance ratings for each discriminatory maneuver

Region	Diagnosis: discriminatory maneuver	Inter-rater reliability (ICC)	Mean (sd) performance	Percent perfect score
Back	L5 radiculopathy (Radic): Straight leg raise	.81	1.62 (.64)	64 %
	Sacroiliac dysfunction (SI): FAbER maneuver	.85	0.84 (.86)	24 %
	Lumbar stenosis (LS): No maneuver	--	--	--
Shoulder	Rotator cuff impingement (Impingement): Jobe test OR Neer test OR Hawkin test	.69	1.58 (.56)	53 %
	Glenohumeral arthritis (GH arthritis): Assessment for crepitation and pain reproduction with glenohumeral grind	.76	0.26 (.57)	4 %
	Adhesive capsulitis (Ad Cap): Comparison of active versus passive range of motion	--	--	--
Knee	ACL tear (ACL): anterior drawer OR Lachman maneuver	.87	1.60 (.51)	56 %
	Osteoarthritis (Knee OA) (palpation for compartmental crepitus)	.87	0.82 (.86)	29 %
	Patellofemoral syndrome (PF) (patellar grind maneuver or observation for abnormal patellar tracking)	--	--	--

**Fig. 1:**
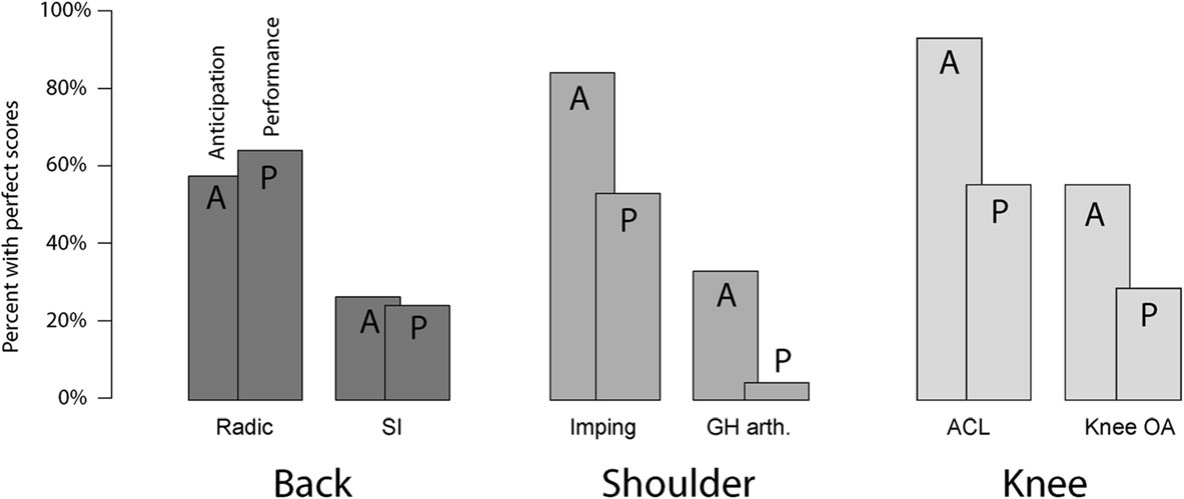
Percent of students with perfect scores on anticipation (A) and performances (P) of observable maneuvers to diagnose specific disorders

Students were better at hypothesis-driven reasoning than they were at performing maneuvers. For the shoulder and knee stations, more students demonstrated perfect anticipation than perfect performance (Fig. 1). Paired t-tests of scores showed significantly lower P scores than A scores for SI (t(44) = 2.28, *p* < .05), GH Arthritis (t(44) = 3.82, *p* < 0.001), ACL (t(44) = 3.45, *p* < 0.0025), and Knee OA (t(44) = 2.94, *p* < 0.01). Interestingly, performance scores were higher than anticipation scores for Radic (t(44) = -3.20, *p* < 0.005) and no significant difference was observed for Impingement (t(44) = 1.26, n.s.).

Students who more accurately anticipated the discriminatory findings also performed the associated maneuvers better, with the exception of the ACL maneuver. The correlations of A and P for Radic (*r* = .45, *p* < .0025), SI (*r* = .67, *p* < .0001), Impingement (*r* = .38, *p* < .025), GH Arthritis (*r* = .32, *p* < .05), and Knee OA (*r* = .33, < .05) were positive and statistically significant. For ACL, A and P scores were not correlated (*r* = .05, n.s.) largely due to the lack of variance in A scores: 93 % of students anticipated ACL perfectly.

Students tended to select the correct diagnosis (Back 87 % correct, Shoulder 82 % correct, Knee 93 % correct), but student anticipation and performance were not predictive (Table 3). For the back and knee cases, estimated log odds for both A and P scores predicting a correct diagnosis for each region did not differ significantly from zero. For the shoulder case, only the GH arthritis maneuver differed from zero with a p-value less than .10 (log odds = 1.54, z(37) = 1.74, *p* < .10) and the other three parameters were not significant.

**Table 3:** For each maneuver, the number and percent of students who diagnosed the case correctly organized by perfect and imperfect score on anticipation (A)

Back					
*Disc*	imperfect A	perfect A	*SI*	imperfect A	perfect A
Imperfect P	5/7 (71 %)	4/6 (67 %)	imperfect P	21/26 (81 %)	2/2 (100 %)
Perfect P	8/9 (89 %)	17/17 (100 %)	perfect P	2/2 (100 %)	9/9 (100 %)
Model fit:	*X*2 (36) = 25.9	n.s.		*X*2 (36) = 28.9	n.s.
Shoulder					
*Imping*	imperfect A	perfect A	*GH Arthrit*	imperfect A	perfect A
Imperfect P	5/5 (100 %)	9/13 (69 %)	imperfect P	20/26 (77 %)	13/14 (93 %)
Perfect P	2/2 (100 %)	19/22 (86 %)	perfect P	1/1 (100 %)	1/1 (100 %)
Model fit:				*X*2 (42) = 41.9	n.s.
Knee					
*ACL*	imperfect A	perfect A	*Knee OA*	imperfect A	perfect A
Imperfect P	1/1 (100 %)	17/19 (89 %)	imperfect P	15/18 (83 %)	14/14 (100 %)
Perfect P	2/2 (100 %)	22/23 (96 %)	perfect P	2/2 (100 %)	11/11 (100 %)
Model fit:	*X*2 (42) = 38.3	n.s.		*X*2 (42) = 17.8	n.s.

No discernable differences in anticipation, performance, or diagnostic ability were observed between students who planned on specializing in a musculoskeletal field, a primary care field, or other specialties; or upon controlling for demographic variables and previous elective musculoskeletal experiences.

## Discussion

We developed an OSCE, grounded in educational theory, designed to not only assess students’ abilities to perform musculoskeletal examinations, but to explore the processes linking knowledge and skill performance. This was achieved through limiting the number of diagnoses to be considered per region, and incorporating a specific pre-encounter anticipation step for each possible diagnosis. Using this construction, we were able to demonstrate that student performance on the OSCE appeared to be limited by their ability to perform the maneuvers and by their ability to use the information acquired by the maneuvers in their diagnostic reasoning. Because their anticipation of findings was better than their examination performance, we hypothesize that it is the skills themselves which pose the greatest challenge.

A substantial percentage of the graduating students in our sample were not able to perform core examination skills needed to diagnose common disorders of the shoulder, back and knee. Three maneuvers in particular (GH Arthritis, Knee OA, and SI) were very difficult for learners, with only 4 to 26 % of students performing the maneuvers perfectly. Students in our cohort had learned about all the clinical disorders and had learned the relevant maneuvers at least 1 year prior; thus the inability to perform was not due to lack of training. We hypothesize that their inability to retain these skills might have been due to lack of practice in a real clinical setting.

We observed that students’ anticipation ability was related to their performance, with students who correctly anticipated expected physical examination findings more able to perform the associated diagnostic maneuver correctly. This suggests that some students have an understanding of the relationship of the physical exam and its findings to the differential diagnosis for a given case. A larger sample would be needed to test this hypothesis more directly. We are currently performing a follow-up study more closely examining the importance of this link between knowledge and skill.

Finally, because neither students’ anticipation nor skills performance appeared related to diagnostic accuracy, there seems to be a deficit in students’ ability to use the information gained from the physical examination in their clinical reasoning. One possibility is that students are relying on cues about appropriate maneuvers and correct diagnoses from details such as the case description, simulated patient behavior, or meta-task information about educators’ intentions. This suggests that students are applying heuristics (“rules of thumb”) about the type of case presented rather than incorporating clinical exam data obtained from the case. Students who rely on heuristics will perform well on knowledge tests despite having poor clinical examination skills. The Freedman & Bernstein test [7] favors heuristics-based thinking with items asking, after brief case descriptions, “[w]hat diagnosis must be considered?” and “[w]hat are the three most common diagnoses?” Our results suggest that students who perform well on such items are not necessarily more capable of examining and diagnosing a patient’s disorder, and that written test scores will overestimate students’ clinical ability.

If the link between knowledge and skills proves to be an important predictor of diagnostic accuracy, a simulation practice-based approach to teaching these skills may prove the most effective. While knowledge of underlying causes of various injuries is crucial for diagnosis, knowledge is not sufficient for physical examination skill. Further, while students show great deficits in physical examination skills, those who anticipated findings were able to better perform the relevant diagnostic examination maneuvers. More OSCEs such as the one we have developed which allow students to pre-plan an exam may be necessary to improve student performance on these sorts of cases.

There are several limitations to this study. The small sample may not generalize to other schools or to other cohorts within our own school. The sample size was not sensitive enough to detect differences between different types of trainees (gender, future specialty, previous experience) and so were not explored statistically here. The correlation between anticipation and performance was merely correlational, and so no causal assertion can definitively be made about any improvement in clinical performance due to the adoption of a HDPE framework. The cases were simulated based on common clinical scenarios, so students may have been able to use common sense, book knowledge, and prior experience to make educated guesses at the correct diagnosis without reference to physical examination findings. Further work is needed to clarify the relationship between students’ information-gathering and diagnostic reasoning during a musculoskeletal physical examination.

## Conclusion

Given the ubiquity of musculoskeletal disorders, and the significant gaps in medical students’ knowledge and skills necessary for their diagnosis and treatment, it is important to understand how to teach and assess the relevant physical examination maneuvers, along with how to interpret the results. Our findings suggest that students’ primary deficits are in their ability to perform examination maneuvers and in their ability to use the results to inform their diagnosis. Further work is needed to design the appropriate education interventions and assessments.

## Additional file

Additional file 1: The Appendix document provides supplementary documentation detailing how the OSCE was performed. Specifically, this includes. 1. The instructions provided to students. 2. A sample vignette provided to students rotating through the “Shoulder” station of the OSCE. 3. A sample checklist for an instructor rating the “Shoulder” station. 4. The scoring rubric used to score the multiple components. (DOCX 95 kb)

## Abbreviations

A: Anticipation score
HDPE: Hypothesis-driven physical examination
ICC: Intraclass correlation coefficient
OSCE: Objective structured clinical examination
P: Performance score
SP: Standardized patient
