# Supplementary material for: Assessing musculoskeletal examination skills and diagnostic reasoning of 4th year medical students using a novel objective structured clinical exam
Source: BMC Med Educ. 2016 Oct 14;16:268. doi: 10.1186/s12909-016-0780-4 (PMC5065081; doi:10.1186/s12909-016-0780-4)
Supplement: Additional file 1: — The Appendix document provides supplementary documentation detailing how the OSCE was performed. Specifically, this includes. 1. The instructions provided to students. 2. A sample vignette provided to students rotating through the “Shoulder” station of the OSCE. 3. A sample checklist for an instructor rating the “Shoulder” station. 4. The scoring rubric used to score the multiple components. (DOCX 95 kb) [file 12909_2016_780_MOESM1_ESM.docx]

**Appendix: Instructions for students**

1. You will be rotating through 3 stations, each with a standardized patient (SP)
2. Prior to each station, you will be handed a clinical case, along with three suggested diagnoses for each case.
3. Before examining the patient, write down what exam findings you would anticipate for each possible diagnosis, especially findings that will help differentiate between one diagnosis and the others.
4. Go in and perform a directed exam to distinguish between your diagnostic considerations. You may do a full exam if you prefer, but we are particularly interested in the maneuvers you feel are most helpful in making your diagnosis.
5. Feel free to talk while you are examining the patient, so your instructor can know what you are doing. He/she will also provide you with any pertinent positive/negative findings based on the maneuvers you perform, if they cannot be simulated by the patient.
6. Write down your preferred diagnosis and what maneuvers/findings helped you come to the diagnosis
7. Your instructor will then provide you feedback
8. Rotate to the next station

Sample timeline: 20:00 minutes

0-5:00 – review vignette and write down anticipated findings

5:00-15:00 – examine patient, document findings and preferred diagnosis

15:00-20:00 – instructor feedback

Rotate to next station

**Appendix: Sample Vignette for Student- Shoulder**

**Complaint: Right shoulder pain**

A 55 year old hairdresser presents for evaluation of right shoulder pain noticeable when she cuts hair. She feels it along the side of her shoulder and is relieved when she rests her arms at her sides.

Your differential diagnosis includes adhesive capsulitis, glenohumeral arthritis, and rotator cuff tendinopathy with impingement.

In anticipation of your physical exam of the shoulder, list several positive signs associated with each diagnostic hypothesis, and mark which one (s) you think are most important in making the diagnosis.

| **Adhesive capsulitis** | **Glenohumeral arthritis** | **Rotator cuff tendinopathy with impingement** |
| --- | --- | --- |
|  |  |  |

**Please answer the following questions, using this scale:**

**1 – Strongly disagree 2-Disagree 3-Neutral 4-Agree 5-Strongly Agree**

1. Right now, I can perform a focused physical exam that can diagnose:
   1. Adhesive capsulitis 1 2 3 4 5
   2. Glenohumeral arthritis 1 2 3 4 5
   3. Rotator cuff tendinopathy 1 2 3 4 5
2. I anticipate that in my future career, I will need the skills to perform a focused physical exam to diagnose:
   1. Adhesive capsulitis 1 2 3 4 5
   2. Glenohumeral arthritis 1 2 3 4 5
   3. Rotator cuff tendinopathy 1 2 3 4 5

Now examine the patient, **focusing on distinguishing between your three possible diagnoses.**

After examining the patient, select your favored diagnosis. List the exam findings that made you choose this diagnosis:

| **[ ] Adhesive capsulitis** |  |
| --- | --- |
| **[ ] Glenohumeral arthritis** |  |
| **[ ] Rotator cuff tendinopathy with impingement** |  |
| **Findings of unclear significance** |  |

**Appendix: Sample Instructor checklist – Shoulder**

| **Exam** | **All done** | | | **Not done** | **Incomplete/inaccurate (describe)** | | |  |
| --- | --- | --- | --- | --- | --- | --- | --- | --- |
| **Examines shoulder** (symmetry, shoulder height, muscle bulk sulcus sign) |  | | |  |  | | |  |
| **Palpates for tenderness**   - AC joint - Subdeltoid bursa - Supraspinatus - Infraspinatus |  | | |  |  | | |  |
| **Active ROM**  -flexion/extension  -abduction/adduction  -internal/external rotation |  | | |  |  | | |  |
| **Passive ROM**  -flexion/extension  -abduction/adduction  -internal/external rotation |  | | |  |  | | |  |
| **Tested infraspinatus strength**  -elbows at side, flexed, external rotation against resistance |  | | |  |  | | |  |
| **Tested subscapularis strength**   1. Lift off – hand on small of back, elevate hand at level of shoulder 2. Belly press: hand on belly, internal rotation of shoulder |  | | |  |  | | |  |
| **Neer’s impingement sign**  -arm internally rotated  -passive flexion to 180° |  | | |  |  | | |  |
| **Hawkin’s impingement**  -arm passively abducted to 90°  -flexed to 90°  -internally rotated | |  | |  |  | | |  |
| **Empty can**  -Shoulder abducted to 90°  -Flexed to 70°  -Internally rotated  -Resistance of downward pressure  -Asks if symptoms reproduced | |  |  | | |  |  |  |
| **Drop arm test**  -arm abducted to 180° (?)  -slowly lowering to 0°  -notes whether test is positive (inability to hold arm abducted) | |  |  | | |  |  |  |
| **Glenohumeral crepitation**  -inferior compression of shoulder joint  -passive flexion/extension of shoulder | |  |  | | |  | |  |
| **Glenohumeral grind** | |  |  | | |  | |  |
| **Shoulder apprehension** | |  |  | | |  | |  |
| **Biceps tendon testing** | |  |  | | |  | |  |
| **Notes:** | | | | | | | |  |

**Appendix: Scoring Rubric**

1. Back
   1. Disc herniation (correct)
      1. Anticipated exam findings: 0 = no, 1 = yes
         1. Answers accepted: Straight leg raise
      2. Self assessment on ability to examine: 1-5 (Likert)
      3. Anticipated future utility: 1-5 (Likert)
      4. Performed: 0 = no, 1 = partial, 2 = full
         1. Partial: Unilateral
   2. Lumbar stenosis
      1. Anticipated: 0 = no, 1 = yes
         1. Answers accepted: No PE findings OR improvement with forward flexion
      2. Self assessment on ability to examine: 1-5 (Likert)
      3. Anticipated future utility: 1-5 (Likert)
   3. Sacroiliac dysfunction
      1. Anticipated: 0 = no, 1 = yes
         1. Answers accepted: FABER
      2. Self assessment on ability to examine: 1-5 (Likert)
      3. Anticipated future utility: 1-5 (Likert)
      4. Performed: 0 = no, 1 = partial, 2 = full
         1. Partial: no hip pressure
   4. Correct diagnosis selected? 0 = no, 1 = yes
   5. Correct explanation listed? 0 = no, 1 = yes
2. Shoulder
   1. Impingement (correct)
3. Anticipated: 0 = no, 1 = yes
   - - 1. Answers accepted: Neer’s, Hawkins, empty can, painful arc
     1. Self assessment on ability to examine: 1-5 (Likert)
     2. Anticipated future utility: 1-5 (Likert)
4. Performed: 0 = no, 1 = partial, 2 = full
   1. Partial: Empty can without resistance; unilateral; full abduction
   2. Adhesive capsulitis
5. Anticipated: 0 = no, 1 = yes
   - - 1. Answers accepted: restricted passive = active ROM
     1. Self assessment on ability to examine: 1-5 (Likert)
     2. Anticipated future utility: 1-5 (Likert)
6. Performed: 0 = no, 1 = partial, 2 = full
   1. Partial: unilateral
   2. Glenohumeral arthritis
7. Anticipated: 0 = no, 1 = yes
   - - 1. Answers accepted: glenohumeral crepitation
     1. Self assessment on ability to examine: 1-5 (Likert)
     2. Anticipated future utility: 1-5 (Likert)
8. Performed: 0 = no, 1 = partial, 2 = full
   1. Partial: unilateral
   2. Correct diagnosis selected? 0 = no, 1 = yes
   3. Correct explanation listed? 0 = no, 1 = yes
9. Knee
   1. ACL (correct)
10. Anticipated: 0 = no, 1 = yes
    - - 1. Answers accepted: Lachman, drawer
      1. Self assessment on ability to examine: 1-5 (Likert)
      2. Anticipated future utility: 1-5 (Likert)
11. Performed: 0 = no, 1 = partial, 2 = full
    1. No: unilateral
    2. Partial: wrong angle; lack of foot stabilization
    3. OA
12. Anticipated: 0 = no, 1 = yes
    - - 1. Answers accepted: patellofemoral crepitation, (bony enlargement)
      1. Self assessment on ability to examine: 1-5 (Likert)
      2. Anticipated future utility: 1-5 (Likert)
13. Performed: 0 = no, 1 = partial, 2 = full
    1. Partial: unilateral
    2. Patellofemoral
14. Anticipated: 0 = no, 1 = yes
    - - 1. Answers accepted: patellar grind, patellar tracking
      1. Self assessment on ability to examine: 1-5 (Likert)
      2. Anticipated future utility: 1-5 (Likert)
15. Performed: 0 = no, 1 = partial, 2 = full
    1. Partial: unilateral, incorrect technique
    2. Correct diagnosis selected? 0 = no, 1 = yes
    3. Correct explanation listed? 0 = no, 1 = yes
